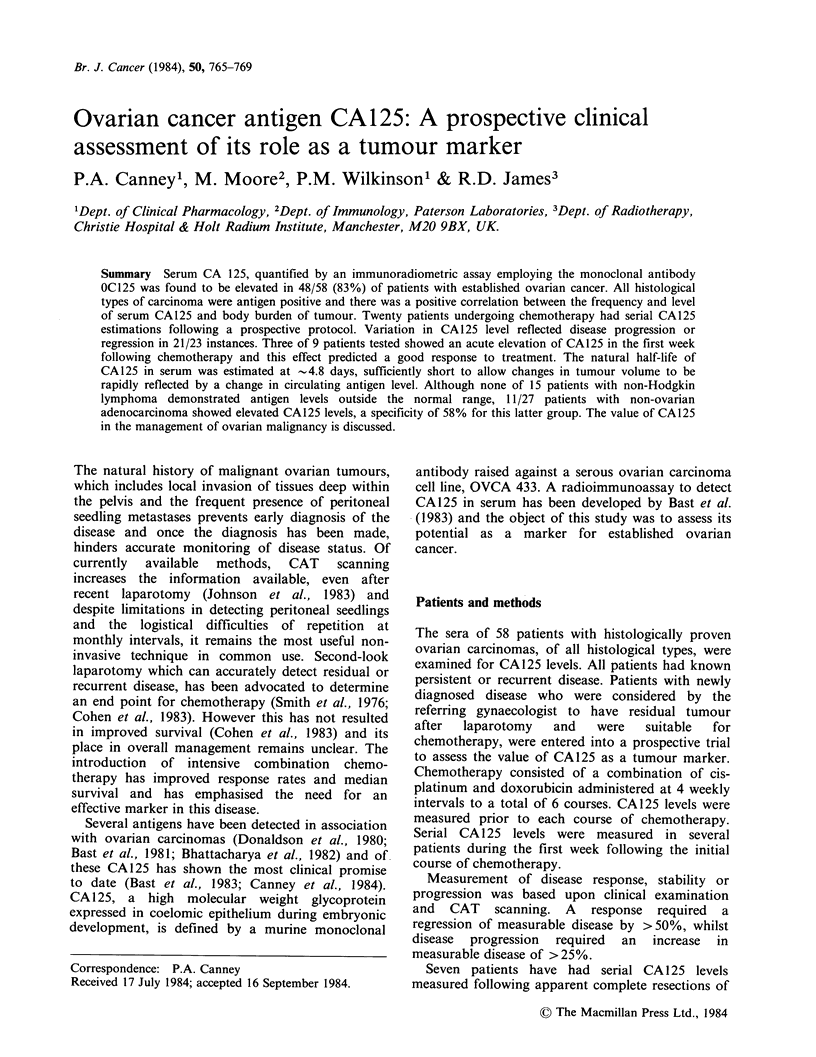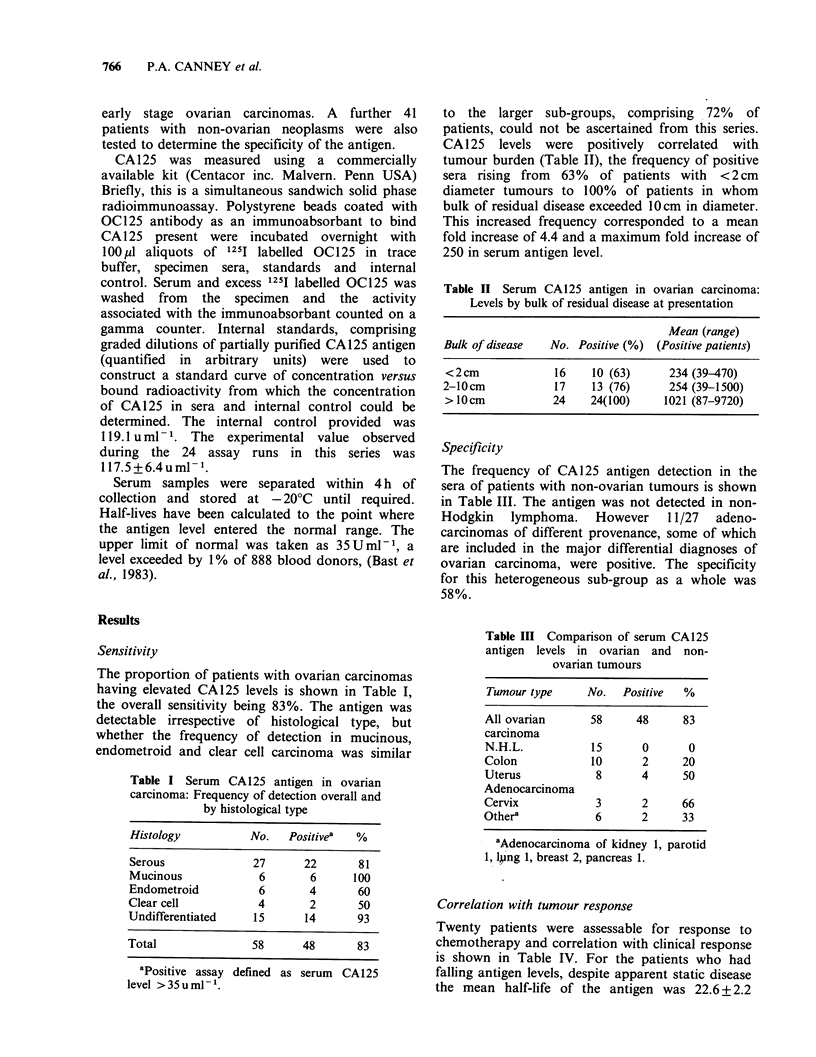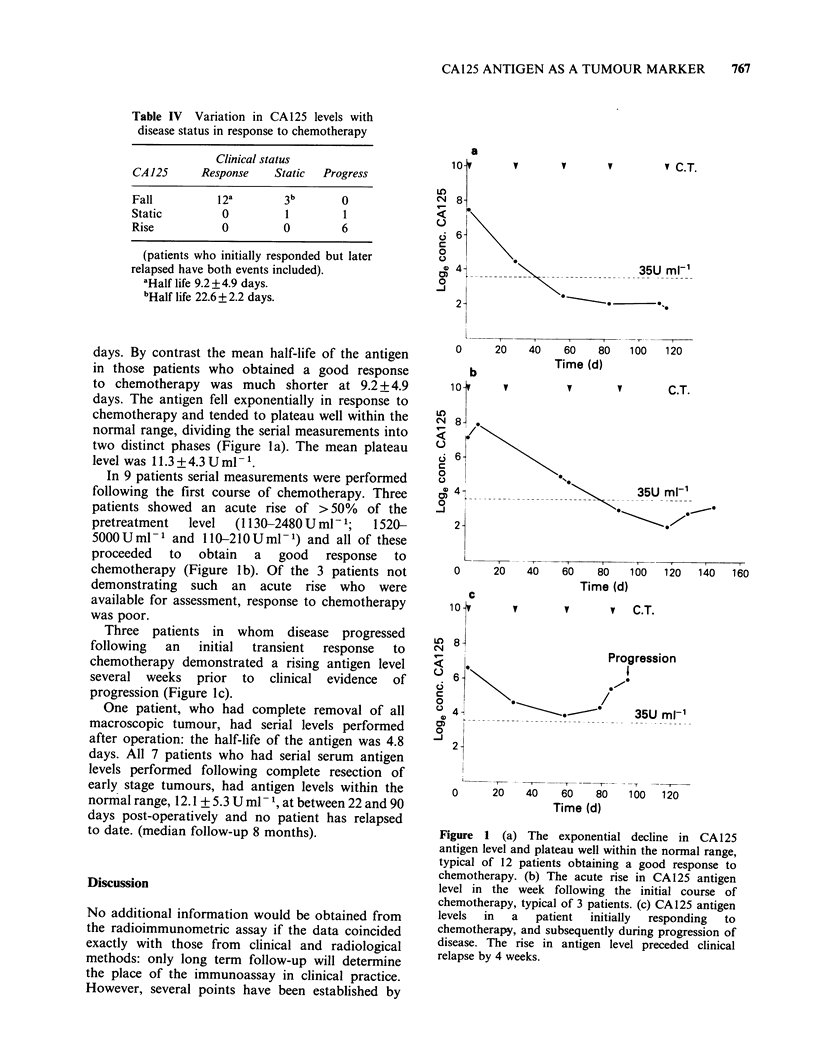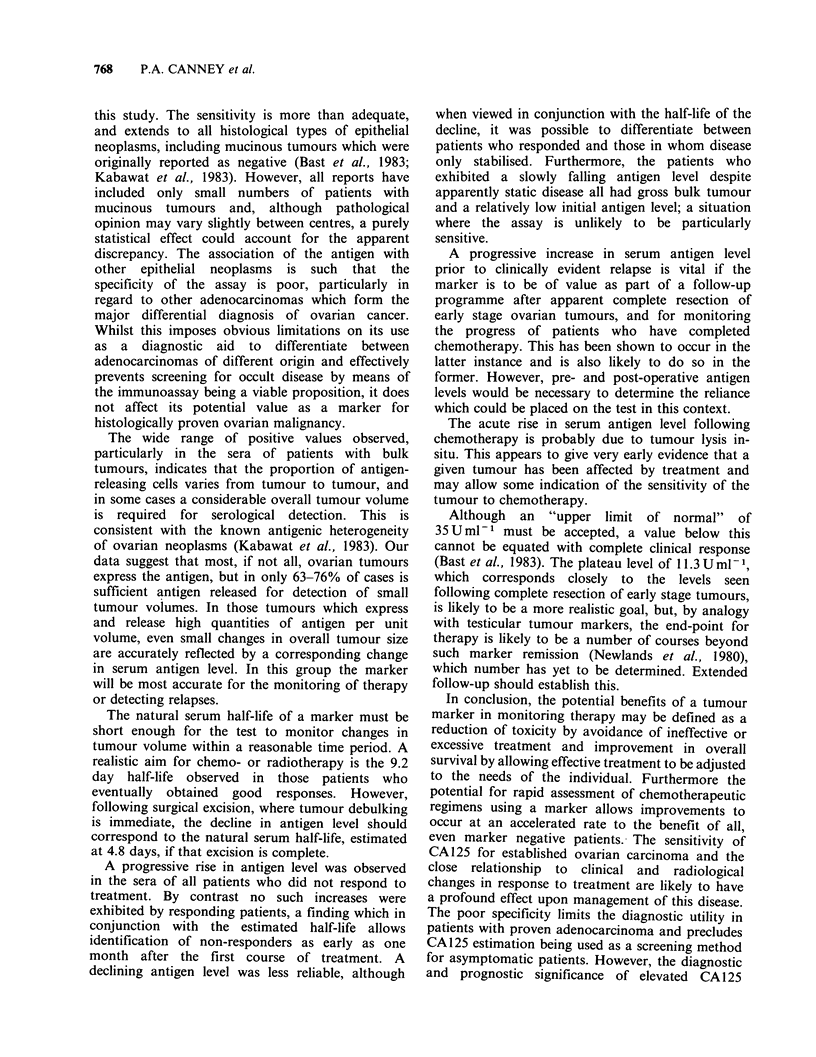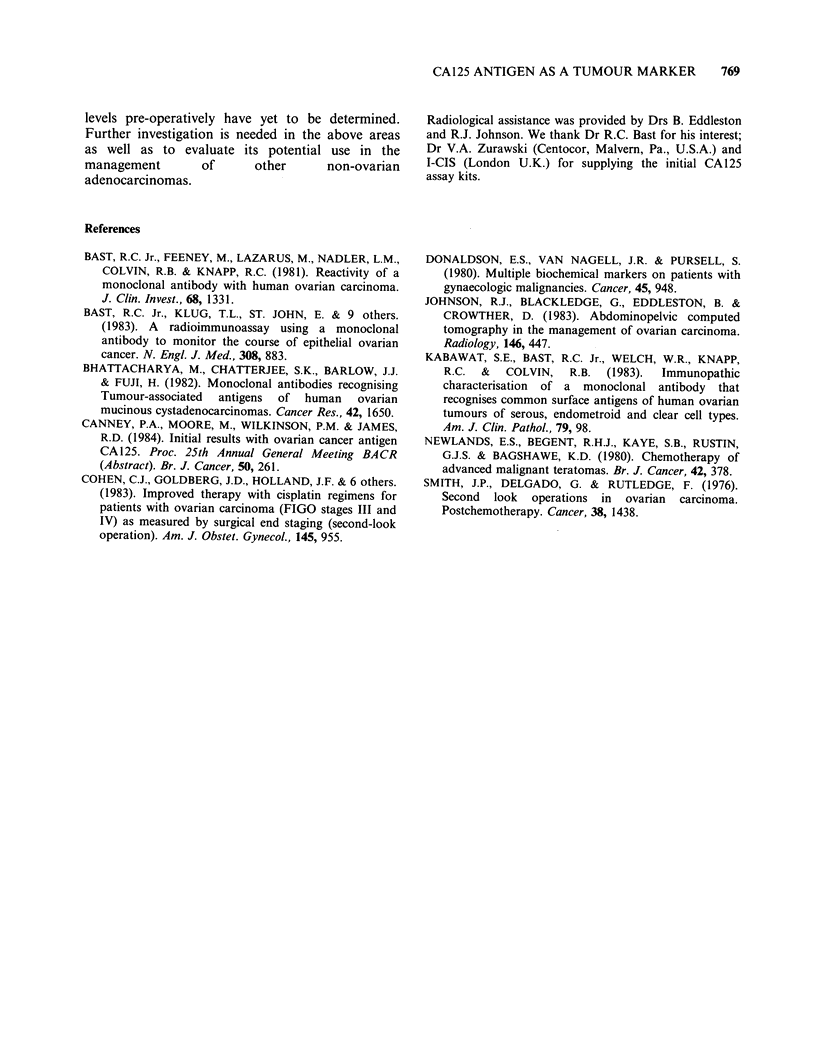# Ovarian cancer antigen CA125: a prospective clinical assessment of its role as a tumour marker.

**DOI:** 10.1038/bjc.1984.254

**Published:** 1984-12

**Authors:** P. A. Canney, M. Moore, P. M. Wilkinson, R. D. James

## Abstract

Serum CA 125, quantified by an immunoradiometric assay employing the monoclonal antibody 0C125 was found to be elevated in 48/58 (83%) of patients with established ovarian cancer. All histological types of carcinoma were antigen positive and there was a positive correlation between the frequency and level of serum CA125 and body burden of tumour. Twenty patients undergoing chemotherapy had serial CA125 estimations following a prospective protocol. Variation in CA125 level reflected disease progression or regression in 21/23 instances. Three of 9 patients tested showed an acute elevation of CA125 in the first week following chemotherapy and this effect predicted a good response to treatment. The natural half-life of CA125 in serum was estimated at approximately 4.8 days, sufficiently short to allow changes in tumour volume to be rapidly reflected by a change in circulating antigen level. Although none of 15 patients with non-Hodgkin lymphoma demonstrated antigen levels outside the normal range, 11/27 patients with non-ovarian adenocarcinoma showed elevated CA125 levels, a specificity of 58% for this latter group. The value of CA125 in the management of ovarian malignancy is discussed.


*Br. J. Cancer* (1984), **50**, 765–769

### Ovarian cancer antigen CA125: A prospective clinical assessment of its role as a tumour marker

P.A. Canney[1], M. Moore[2], P.M. Wilkinson[1] & R.D. James[3]

[1]*Dept. of Clinical Pharmacology*, [2]*Dept. of Immunology, Paterson Laboratories*, [3]*Dept. of Radiotherapy, Christie Hospital & Holt Radium Institute, Manchester, M20 9BX, UK.*

**Summary** Serum CA 125, quantified by an immunoradiometric assay employing the monoclonal antibody 0C125 was found to be elevated in 48/58 (83%) of patients with established ovarian cancer. All histological types of carcinoma were antigen positive and there was a positive correlation between the frequency and level of serum CA125 and body burden of tumour. Twenty patients undergoing chemotherapy had serial CA125 estimations following a prospective protocol. Variation in CA125 level reflected disease progression or regression in 21/23 instances. Three of 9 patients tested showed an acute elevation of CA125 in the first week following chemotherapy and this effect predicted a good response to treatment. The natural half-life of CA125 in serum was estimated at ~4.8 days, sufficiently short to allow changes in tumour volume to be rapidly reflected by a change in circulating antigen level. Although none of 15 patients with non-Hodgkin lymphoma demonstrated antigen levels outside the normal range, 11/27 patients with non-ovarian adenocarcinoma showed elevated CA125 levels, a specificity of 58% for this latter group. The value of CA125 in the management of ovarian malignancy is discussed.

The natural history of malignant ovarian tumours, which includes local invasion of tissues deep within the pelvis and the frequent presence of peritoneal seedling metastases prevents early diagnosis of the disease and once the diagnosis has been made, hinders accurate monitoring of disease status. Of currently available methods, CAT scanning increases the information available, even after recent laparotomy (Johnson *et al.*, 1983) and despite limitations in detecting peritoneal seedlings and the logistical difficulties of repetition at monthly intervals, it remains the most useful non-invasive technique in common use. Second-look laparotomy which can accurately detect residual or recurrent disease, has been advocated to determine an end point for chemotherapy (Smith *et al.*, 1976; Cohen *et al.*, 1983). However this has not resulted in improved survival (Cohen *et al.*, 1983) and its place in overall management remains unclear. The introduction of intensive combination chemotherapy has improved response rates and median survival and has emphasised the need for an effective marker in this disease.

Several antigens have been detected in association with ovarian carcinomas (Donaldson *et al.*, 1980; Bast *et al.*, 1981; Bhattacharya *et al.*, 1982) and of these CA125 has shown the most clinical promise to date (Bast *et al.*, 1983; Canney *et al.*, 1984). CA125, a high molecular weight glycoprotein expressed in coelomic epithelium during embryonic development, is defined by a murine monoclonal antibody raised against a serous ovarian carcinoma cell line, OVCA 433. A radioimmunoassay to detect CA125 in serum has been developed by Bast *et al.* (1983) and the object of this study was to assess its potential as a marker for established ovarian cancer.

Correspondence: P.A. Canney
Received 17 July 1984; accepted 16 September 1984.

### Patients and methods

The sera of 58 patients with histologically proven ovarian carcinomas, of all histological types, were examined for CA125 levels. All patients had known persistent or recurrent disease. Patients with newly diagnosed disease who were considered by the referring gynaecologist to have residual tumour after laparotomy and were suitable for chemotherapy, were entered into a prospective trial to assess the value of CA125 as a tumour marker. Chemotherapy consisted of a combination of cis-platinum and doxorubicin administered at 4 weekly intervals to a total of 6 courses. CA125 levels were measured prior to each course of chemotherapy. Serial CA125 levels were measured in several patients during the first week following the initial course of chemotherapy.

Measurement of disease response, stability or progression was based upon clinical examination and CAT scanning. A response required a regression of measurable disease by >50%, whilst disease progression required an increase in measurable disease of >25%.

Seven patients have had serial CA125 levels measured following apparent complete resections of

© The Macmillan Press Ltd., 1984

early stage ovarian carcinomas. A further 41 patients with non-ovarian neoplasms were also tested to determine the specificity of the antigen.

CA125 was measured using a commercially available kit (Centacor inc. Malvern. Penn USA) Briefly, this is a simultaneous sandwich solid phase radioimmunoassay. Polystyrene beads coated with OC125 antibody as an immunoabsorbant to bind CA125 present were incubated overnight with 100 $\mu$l aliquots of $^{125}$I labelled OC125 in trace buffer, specimen sera, standards and internal control. Serum and excess $^{125}$I labelled OC125 was washed from the specimen and the activity associated with the immunoabsorbant counted on a gamma counter. Internal standards, comprising graded dilutions of partially purified CA125 antigen (quantified in arbitrary units) were used to construct a standard curve of concentration *versus* bound radioactivity from which the concentration of CA125 in sera and internal control could be determined. The internal control provided was 119.1 u ml$^{-1}$. The experimental value observed during the 24 assay runs in this series was 117.5 $\pm$ 6.4 u ml$^{-1}$.

Serum samples were separated within 4 h of collection and stored at $-20$°C until required. Half-lives have been calculated to the point where the antigen level entered the normal range. The upper limit of normal was taken as 35 U ml$^{-1}$, a level exceeded by 1% of 888 blood donors, (Bast *et al.*, 1983).

### Results

### *Sensitivity*

The proportion of patients with ovarian carcinomas having elevated CA125 levels is shown in Table I, the overall sensitivity being 83%. The antigen was detectable irrespective of histological type, but whether the frequency of detection in mucinous, endometroid and clear cell carcinoma was similar to the larger sub-groups, comprising 72% of patients, could not be ascertained from this series. CA125 levels were positively correlated with tumour burden (Table II), the frequency of positive sera rising from 63% of patients with <2 cm diameter tumours to 100% of patients in whom bulk of residual disease exceeded 10 cm in diameter. This increased frequency corresponded to a mean fold increase of 4.4 and a maximum fold increase of 250 in serum antigen level.

**Table I** Serum CA125 antigen in ovarian carcinoma: Frequency of detection overall and by histological type

| *Histology* | *No.* | *Positive*[a] | % |
|---|---|---|---|
| Serous | 27 | 22 | 81 |
| Mucinous | 6 | 6 | 100 |
| Endometroid | 6 | 4 | 60 |
| Clear cell | 4 | 2 | 50 |
| Undifferentiated | 15 | 14 | 93 |
| Total | 58 | 48 | 83 |

[a]Positive assay defined as serum CA125 level >35 u ml$^{-1}$.

**Table II** Serum CA125 antigen in ovarian carcinoma: Levels by bulk of residual disease at presentation

| *Bulk of disease* | *No.* | *Positive* (%) | *Mean* (*range*) (*Positive patients*) |
|---|---|---|---|
| <2 cm | 16 | 10 (63) | 234 (39–470) |
| 2–10 cm | 17 | 13 (76) | 254 (39–1500) |
| >10 cm | 24 | 24(100) | 1021 (87–9720) |

### *Specificity*

The frequency of CA125 antigen detection in the sera of patients with non-ovarian tumours is shown in Table III. The antigen was not detected in non-Hodgkin lymphoma. However 11/27 adenocarcinomas of different provenance, some of which are included in the major differential diagnoses of ovarian carcinoma, were positive. The specificity for this heterogeneous sub-group as a whole was 58%.

**Table III** Comparison of serum CA125 antigen levels in ovarian and non-ovarian tumours

| *Tumour type* | *No.* | *Positive* | % |
|---|---|---|---|
| All ovarian carcinoma | 58 | 48 | 83 |
| N.H.L. | 15 | 0 | 0 |
| Colon | 10 | 2 | 20 |
| Uterus Adenocarcinoma | 8 | 4 | 50 |
| Cervix | 3 | 2 | 66 |
| Other[a] | 6 | 2 | 33 |

[a]Adenocarcinoma of kidney 1, parotid 1, lung 1, breast 2, pancreas 1.

### *Correlation with tumour response*

Twenty patients were assessable for response to chemotherapy and correlation with clinical response is shown in Table IV. For the patients who had falling antigen levels, despite apparent static disease the mean half-life of the antigen was 22.6 $\pm$ 2.2

**Table IV** Variation in CA125 levels with disease status in response to chemotherapy

| *CA125* | *Clinical status* *Response* | *Static* | *Progress* |
|---|---|---|---|
| Fall | 12[a] | 3[b] | 0 |
| Static | 0 | 1 | 1 |
| Rise | 0 | 0 | 6 |

(patients who initially responded but later relapsed have both events included).
[a]Half life 9.2±4.9 days.
[b]Half life 22.6±2.2 days.

days. By contrast the mean half-life of the antigen in those patients who obtained a good response to chemotherapy was much shorter at 9.2±4.9 days. The antigen fell exponentially in response to chemotherapy and tended to plateau well within the normal range, dividing the serial measurements into two distinct phases (Figure 1a). The mean plateau level was 11.3±4.3 U $ml^{-1}$.

In 9 patients serial measurements were performed following the first course of chemotherapy. Three patients showed an acute rise of >50% of the pretreatment level (1130–2480 U $ml^{-1}$; 1520–5000 U $ml^{-1}$ and 110–210 U $ml^{-1}$) and all of these proceeded to obtain a good response to chemotherapy (Figure 1b). Of the 3 patients not demonstrating such an acute rise who were available for assessment, response to chemotherapy was poor.

Three patients in whom disease progressed following an initial transient response to chemotherapy demonstrated a rising antigen level several weeks prior to clinical evidence of progression (Figure 1c).

One patient, who had complete removal of all macroscopic tumour, had serial levels performed after operation: the half-life of the antigen was 4.8 days. All 7 patients who had serial serum antigen levels performed following complete resection of early stage tumours, had antigen levels within the normal range, 12.1±5.3 U $ml^{-1}$, at between 22 and 90 days post-operatively and no patient has relapsed to date. (median follow-up 8 months).

### Discussion

No additional information would be obtained from the radioimmunometric assay if the data coincided exactly with those from clinical and radiological methods: only long term follow-up will determine the place of the immunoassay in clinical practice. However, several points have been established by

**Figure 1** (a) The exponential decline in CA125 antigen level and plateau well within the normal range, typical of 12 patients obtaining a good response to chemotherapy. (b) The acute rise in CA125 antigen level in the week following the initial course of chemotherapy, typical of 3 patients. (c) CA125 antigen levels in a patient initially responding to chemotherapy, and subsequently during progression of disease. The rise in antigen level preceded clinical relapse by 4 weeks.

this study. The sensitivity is more than adequate, and extends to all histological types of epithelial neoplasms, including mucinous tumours which were originally reported as negative (Bast *et al.*, 1983; Kabawat *et al.*, 1983). However, all reports have included only small numbers of patients with mucinous tumours and, although pathological opinion may vary slightly between centres, a purely statistical effect could account for the apparent discrepancy. The association of the antigen with other epithelial neoplasms is such that the specificity of the assay is poor, particularly in regard to other adenocarcinomas which form the major differential diagnosis of ovarian cancer. Whilst this imposes obvious limitations on its use as a diagnostic aid to differentiate between adenocarcinomas of different origin and effectively prevents screening for occult disease by means of the immunoassay being a viable proposition, it does not affect its potential value as a marker for histologically proven ovarian malignancy.

The wide range of positive values observed, particularly in the sera of patients with bulk tumours, indicates that the proportion of antigen-releasing cells varies from tumour to tumour, and in some cases a considerable overall tumour volume is required for serological detection. This is consistent with the known antigenic heterogeneity of ovarian neoplasms (Kabawat *et al.*, 1983). Our data suggest that most, if not all, ovarian tumours express the antigen, but in only 63–76% of cases is sufficient antigen released for detection of small tumour volumes. In those tumours which express and release high quantities of antigen per unit volume, even small changes in overall tumour size are accurately reflected by a corresponding change in serum antigen level. In this group the marker will be most accurate for the monitoring of therapy or detecting relapses.

The natural serum half-life of a marker must be short enough for the test to monitor changes in tumour volume within a reasonable time period. A realistic aim for chemo- or radiotherapy is the 9.2 day half-life observed in those patients who eventually obtained good responses. However, following surgical excision, where tumour debulking is immediate, the decline in antigen level should correspond to the natural serum half-life, estimated at 4.8 days, if that excision is complete.

A progressive rise in antigen level was observed in the sera of all patients who did not respond to treatment. By contrast no such increases were exhibited by responding patients, a finding which in conjunction with the estimated half-life allows identification of non-responders as early as one month after the first course of treatment. A declining antigen level was less reliable, although when viewed in conjunction with the half-life of the decline, it was possible to differentiate between patients who responded and those in whom disease only stabilised. Furthermore, the patients who exhibited a slowly falling antigen level despite apparently static disease all had gross bulk tumour and a relatively low initial antigen level; a situation where the assay is unlikely to be particularly sensitive.

A progressive increase in serum antigen level prior to clinically evident relapse is vital if the marker is to be of value as part of a follow-up programme after apparent complete resection of early stage ovarian tumours, and for monitoring the progress of patients who have completed chemotherapy. This has been shown to occur in the latter instance and is also likely to do so in the former. However, pre- and post-operative antigen levels would be necessary to determine the reliance which could be placed on the test in this context.

The acute rise in serum antigen level following chemotherapy is probably due to tumour lysis in-situ. This appears to give very early evidence that a given tumour has been affected by treatment and may allow some indication of the sensitivity of the tumour to chemotherapy.

Although an "upper limit of normal" of $35\,U\,ml^{-1}$ must be accepted, a value below this cannot be equated with complete clinical response (Bast *et al.*, 1983). The plateau level of $11.3\,U\,ml^{-1}$, which corresponds closely to the levels seen following complete resection of early stage tumours, is likely to be a more realistic goal, but, by analogy with testicular tumour markers, the end-point for therapy is likely to be a number of courses beyond such marker remission (Newlands *et al.*, 1980), which number has yet to be determined. Extended follow-up should establish this.

In conclusion, the potential benefits of a tumour marker in monitoring therapy may be defined as a reduction of toxicity by avoidance of ineffective or excessive treatment and improvement in overall survival by allowing effective treatment to be adjusted to the needs of the individual. Furthermore the potential for rapid assessment of chemotherapeutic regimens using a marker allows improvements to occur at an accelerated rate to the benefit of all, even marker negative patients. The sensitivity of CA125 for established ovarian carcinoma and the close relationship to clinical and radiological changes in response to treatment are likely to have a profound effect upon management of this disease. The poor specificity limits the diagnostic utility in patients with proven adenocarcinoma and precludes CA125 estimation being used as a screening method for asymptomatic patients. However, the diagnostic and prognostic significance of elevated CA125

levels pre-operatively have yet to be determined. Further investigation is needed in the above areas as well as to evaluate its potential use in the management of other non-ovarian adenocarcinomas.

Radiological assistance was provided by Drs B. Eddleston and R.J. Johnson. We thank Dr R.C. Bast for his interest; Dr V.A. Zurawski (Centocor, Malvern, Pa., U.S.A.) and I-CIS (London U.K.) for supplying the initial CA125 assay kits.

### References

BAST, R.C. Jr., FEENEY, M., LAZARUS, M., NADLER, L.M., COLVIN, R.B. & KNAPP, R.C. (1981). Reactivity of a monoclonal antibody with human ovarian carcinoma. *J. Clin. Invest.*, **68**, 1331.

BAST, R.C. Jr., KLUG, T.L., ST. JOHN, E. & 9 others. (1983). A radioimmunoassay using a monoclonal antibody to monitor the course of epithelial ovarian cancer. *N. Engl. J. Med.*, **308**, 883.

BHATTACHARYA, M., CHATTERJEE, S.K., BARLOW, J.J. & FUJI, H. (1982). Monoclonal antibodies recognising Tumour-associated antigens of human ovarian mucinous cystadenocarcinomas. *Cancer Res.*, **42**, 1650.

CANNEY, P.A., MOORE, M., WILKINSON, P.M. & JAMES, R.D. (1984). Initial results with ovarian cancer antigen CA125. *Proc. 25th Annual General Meeting BACR (Abstract). Br. J. Cancer*, **50**, 261.

COHEN, C.J., GOLDBERG, J.D., HOLLAND, J.F. & 6 others. (1983). Improved therapy with cisplatin regimens for patients with ovarian carcinoma (FIGO stages III and IV) as measured by surgical end staging (second-look operation). *Am. J. Obstet. Gynecol.*, **145**, 955.

DONALDSON, E.S., VAN NAGELL, J.R. & PURSELL, S. (1980). Multiple biochemical markers on patients with gynaecologic malignancies. *Cancer*, **45**, 948.

JOHNSON, R.J., BLACKLEDGE, G., EDDLESTON, B. & CROWTHER, D. (1983). Abdominopelvic computed tomography in the management of ovarian carcinoma. *Radiology*, **146**, 447.

KABAWAT, S.E., BAST, R.C. Jr., WELCH, W.R., KNAPP, R.C. & COLVIN, R.B. (1983). Immunopathic characterisation of a monoclonal antibody that recognises common surface antigens of human ovarian tumours of serous, endometroid and clear cell types. *Am. J. Clin. Pathol.*, **79**, 98.

NEWLANDS, E.S., BEGENT, R.H.J., KAYE, S.B., RUSTIN, G.J.S. & BAGSHAWE, K.D. (1980). Chemotherapy of advanced malignant teratomas. *Br. J. Cancer*, **42**, 378.

SMITH, J.P., DELGADO, G. & RUTLEDGE, F. (1976). Second look operations in ovarian carcinoma. Postchemotherapy. *Cancer*, **38**, 1438.